# Sensor Selection for Tidal Volume Determination via Linear Regression—Impact of Lasso versus Ridge Regression

**DOI:** 10.3390/s23177407

**Published:** 2023-08-25

**Authors:** Bernhard Laufer, Paul D. Docherty, Rua Murray, Sabine Krueger-Ziolek, Nour Aldeen Jalal, Fabian Hoeflinger, Stefan J. Rupitsch, Leonhard Reindl, Knut Moeller

**Affiliations:** 1 Institute of Technical Medicine (ITeM), Furtwangen University, 78054 Villingen-Schwenningen, Germany; 2 Department of Mechanical Engineering, University of Canterbury, Christchurch 8041, New Zealand; 3 School of Mathematics and Statistics, University of Canterbury, Christchurch 8041, New Zealand; 4 Innovation Center Computer Assisted Surgery (ICCAS), University of Leipzig, 04109 Leipzig, Germany; 5 Department of Microsystems Engineering, University of Freiburg, 79085 Freiburg, Germany

**Keywords:** wearables, smart clothing, sensor selection, linear regression, Lasso, Ridge regression, tidal volume

## Abstract

The measurement of respiratory volume based on upper body movements by means of a smart shirt is increasingly requested in medical applications. This research used upper body surface motions obtained by a motion capture system, and two regression methods to determine the optimal selection and placement of sensors on a smart shirt to recover respiratory parameters from benchmark spirometry values. The results of the two regression methods (Ridge regression and the least absolute shrinkage and selection operator (Lasso)) were compared. This work shows that the Lasso method offers advantages compared to the Ridge regression, as it provides sparse solutions and is more robust to outliers. However, both methods can be used in this application since they lead to a similar sensor subset with lower computational demand (from exponential effort for full exhaustive search down to the order of *O* (*n*^2^)). A smart shirt for respiratory volume estimation could replace spirometry in some cases and would allow for a more convenient measurement of respiratory parameters in home care or hospital settings.

## 1. Introduction

Respiratory volumes and respiration-induced movements of the upper body are connected, and there is a desire to determine tidal volumes via surface motions of the human upper body. Respiration-induced motions were studied as early as 1848 by Sibson et al. [1]. Later, Wade et al. [2] used improved measurement methods and examined the respiratory-induced movements of the upper body, and recently, Laufer et al. [3] analyzed movement parameters of the upper body and their correlations with the respiratory volume in detail.

The pioneers who initiated the research field of determining tidal volumes from upper body movements were Konno and Mead [4]. They investigated the underlying relationships in more detail and published their first studies in the 1960s. The potential of such an approach was recognized, and many other studies followed. Unfortunately, only two measurement methods were able to establish themselves for clinical use and are still used sporadically today. One of these measurement methods is optoelectronic plethysmography [5,6]. The underlying principle of optoelectronic plethysmography is an optical motion tracking system (MoCap). The MoCap system detects respiration-induced movements on the upper body and determines respiration volumes. The disadvantages of an optoelectronic plethysmography are the acquisition costs and the complex procedure of use. It is nevertheless used in very sensitive areas of respiratory monitoring where breathing should not be influenced or impeded in any way by the measurement system—for example, in the respiratory monitoring of premature infants. The second established method is respiratory inductance plethysmography [7,8]. The respiratory inductance plethysmography measures respiratory-induced cross-sectional changes on the upper body inductively and thereby determines tidal volumes. The major disadvantage of respiratory inductance plethysmography is its reduced measurement accuracy [9].

Therefore, despite all efforts to date, tidal volumes are still determined by respiratory flow measurement using spirometers [10,11,12] or body plethysmographs [13,14,15]. For precise clinical respiratory flow measurements, patients under investigation must wear a face mask or breathe through a mouthpiece while the nose is blocked by a nose clip. This can be very inconvenient, especially for long-term measurements, and can falsify the measurement results themselves [16,17].

Hence, there remains a need for alternative methods for measuring respiratory volumes using upper body surface motion. A previous study [3] has shown that upper body movements are in some cases highly correlated with changes in respiratory volumes. This fact can be utilized for the determination of respiratory volumes via upper body movements, and some current approaches used inertial measurement units [18,19,20], others used strain gauges [21,22] or optical encoder systems, as in belts measuring changes in circumferences [23,24,25]. However, a real breakthrough has not been achieved yet despite the potential of new, improved and miniaturized sensors or sensor technologies [26,27]. In particular, the miniaturization and increased precision of sensors improves their integration into garments. In particular, smart shirts are increasingly used in medical diagnostics and therapy monitoring applications, where high accuracy is required [28,29,30]. To date, this has been mostly in the field of cardiovascular monitoring, e.g., heart rate monitoring or respiratory rate monitoring [19,31,32,33,34,35,36]. The Hexoskin Shirt (Montreal, QC, Canada) [37] is a newly launched smart shirt for monitoring vital signs and attempts to determine respiratory minute volumes in addition to common vital signs. However, various studies [38,39,40] have shown that the accuracy of measuring respiratory volumes are still outside the clinically relevant range.

In the development of new smart shirts, it is crucial to determine the optimal number, location and type of sensors to employ in the garment. Data from a MoCap system that tracks respiratory-induced movements at multiple points on the upper body were analyzed with different methods to select the optimal sensor sets for respiratory volume estimation. The MoCap system allows for the determination of various movement parameters of the upper body, such as accelerations, displacements and tilt angles at various surface points. Furthermore, upper body circumferences and local distance changes between the points can be determined [3]. These movement parameters can be measured via corresponding sensors.

In this work, two different regression methods were applied to map different subsets of displacement parameters, generated from the motion capture system data, to tidal volume. A preliminary analysis with five subjects indicated already the capabilities of the regression methods and provided accurate estimates of tidal volume [41]. The regression methods were exemplarily evaluated in this study on the displacement parameters; however, they are fully applicable to the other respiration induced motion parameters of the upper body. Such an analysis is essential to provide confidence in the subset selection for clinical use. Any approach that selects a small number of optimal sensors from a large set of sensors can be supported by the Lasso or Ridge regression, which results in a significant reduction of time and computing power at the cost of some loss in accuracy compared to an exhaustive search.

## 2. Materials and Methods

### 2.1. Measurement Setup

**Figure 1:**
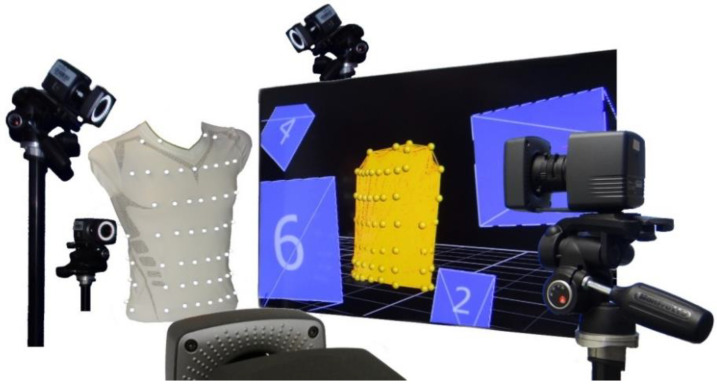
Schematic of the MoCap system. Five of the nine infrared cameras, the compression shirt with MoCap markers and the representation of the MoCap markers by the Vicon Systems on a screen. Figure published in [3].

**Figure 2:**
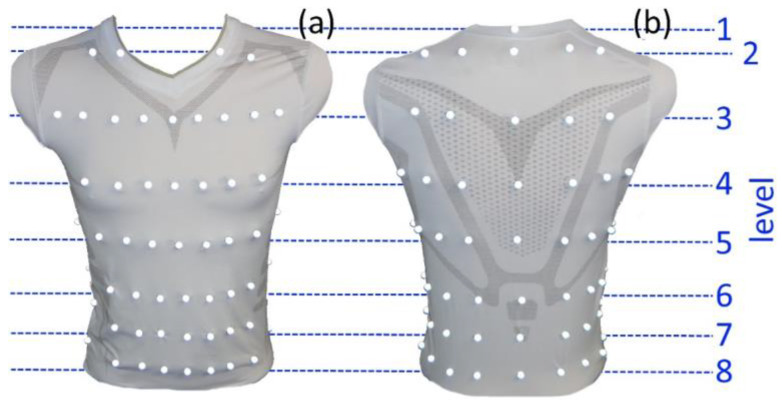
Compression shirt with 102 reflective MoCap markers–ventral view (**a**) and dorsal view (**b**). The markers are arranged in 8 distinct levels. Reference point in dorsal view ((**b**)—level 1) is the highest MoCap marker at the neck of the shirt.

The study was based on the data recorded in Laufer et al. [3], where a motion capture system (MoCap) (Bonita, VICON, Denver, CO, USA) with nine infrared cameras (VICON Bonita B10, firmware version 404) was used to measure the respiratory-induced movements of the human upper body via 102 highly reflective motion capture markers, attached at precise locations on a compression shirt. A schematic of the measurement system is shown in Figure 1. The markers were arranged in 8 different levels, where 48 were located ventrally, 18 laterally and 36 dorsally on the shirt (Figure 2). The highest MoCap marker (collar of the shirt) was located close to the C6 cervical vertebra and serves as a reference point (level 1). Since the cervical spine is barely exposed to respiratory movements, the reference marker can be used together with two other markers along the spine for a movement correction; non-respiratory movements can be eliminated via these 3 reference markers.

As shown in Figure 2, level 2 was approximately located at the level of the thoracic vertebra T1 and at the level of the clavicula, respectively. Level 3 was at the height of T4, while level 4 was at the height of T7, caudally underneath the scapula. Level 5 was at the level of the thoracic vertebra T11, and level 6 was at the height of the lumbar vertebra L1, just at the caudal end of the arcus costalis. Level 7 was at the level of L3, and level 8 was at the height of L5. However, these levels are only approximations and were found to vary depending on the shape of the participants.

Subjects wearing the compression shirt and surrounded by the MoCap cameras performed different breathing patterns while breathing simultaneously through a spirometer (SpiroScout and LFX Software 1.8, Ganshorn Medizin Electronic GmbH, Niederlauer, Germany). The spirometer served as a reference for tidal volume measurement. Flow and volume data were measured with the spirometer at a sampling frequency of 200 Hz. To reduce the dataset slightly, the sampling frequency was set to 40 Hz for the MoCap system. The MoCap system provided the spatial positions of all markers at each time point of the measurement, which were transferred via VICON Nexus software (version 1.8.5.6 1009h, Vicon Motion Systems Ltd., Denver, CO, USA) to MATLAB (R2022a, The MathWorks, Natick, MA, USA) for subsequent calculations.

**Figure 3:**
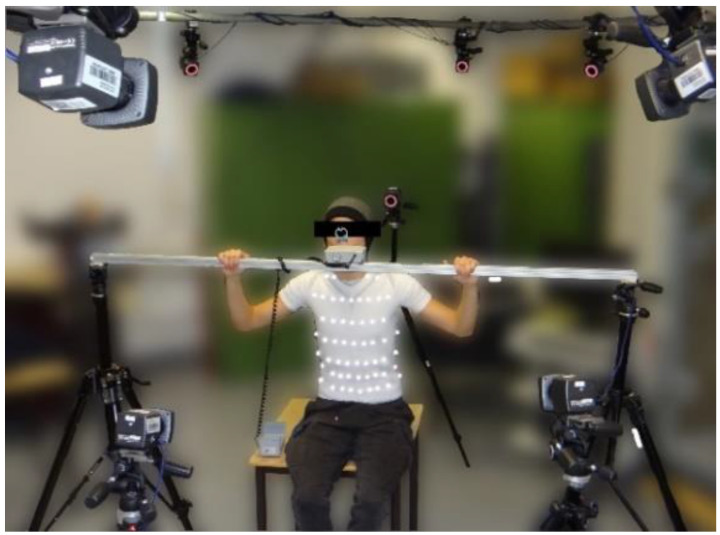
Measurement setup: A subject wearing the compression shirt with MoCap markers, breathing through the spirometer, which was fixed on the rigid mount and surrounded by the MoCap cameras. Figure published in [3].

During measurements, subjects sat as shown in Figure 3. To reduce non-respiratory movements of the upper body, the spirometer was attached to a rigid holder at the level of the subject’s mouth. In this way, movements of the head and upper body were minimal, and the movement data obtained were almost entirely respiratory movements. Additionally, the subjects could rest their arms on that holder. This posture improved the optical detection of lateral markers in MoCap system and was more comfortable as reported by the subjects. To enhance marker detection, the subjects were asked to tie up long hair during the measurement.

### 2.2. Participants and Respiratory Manoeuvres

Ethical approval for this study was obtained from the University of Canterbury Ethics Committee HEC 2019/01/LR-PS and the Furtwangen University Ethics Committee. It was ensured that all measurements were performed in accordance with the principles of the Helsinki Declaration and that subjects were fully informed about the study prior to measurement. In addition, the subjects were informed about any risks, even if the risks associated with these measurements were minor and very unlikely. Signed informed consent was collected from each subject prior to experimentation. The subjects could stop the measurement at any time if they felt the slightest discomfort.

Participants were recruited via an email to the students of the Furtwangen University. Inclusion criteria included lung healthy students. Exclusion criteria included known lung disease, pregnant women and subjects aged under 18.

Three women and thirteen men participated in the measurements. The average height of the subjects was 1.76 ± 0.02 m, the average age was 25.7 ± 2.2 years and the average weight was 69.4 ± 2.0 kg. Further details are listed in Table 1.

**Figure 4:**
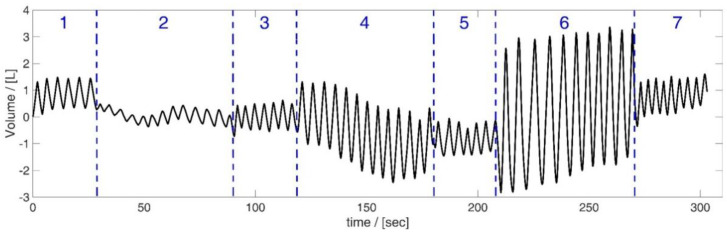
Respiratory patterns shown based on spirometer volume data from subject 5. Breathing of different tidal volumes—shallow (2), medium (4) and maximal breaths (6) between short ranges of normal spontaneous breathing ((1), (3), (5) and (7)). Figure published in [3].

The subjects took different tidal volumes in order to capture as much of the respiratory spectrum as possible. For this purpose, the subjects reduced their breathing activity to a minimum and breathed shallow breaths. Afterwards, the subjects increased the tidal volume beyond the volume of normal spontaneous breathing (but not to the maximum), thus taking medium breaths, and finally, they breathed in and out as far as possible (maximal breaths). As shown in Table 2 and Figure 4, each of these breathing patterns was performed for approximately one minute.

Before and after each breathing pattern, subjects performed 30 s of normal spontaneous breathing to relax and to avoid risk of hyper/hypoventilation. The total measurement time was approximately 5 min, but the exact timing depended on the subject’s breathing rhythm.

### 2.3. Data Processing

**Figure 5:**
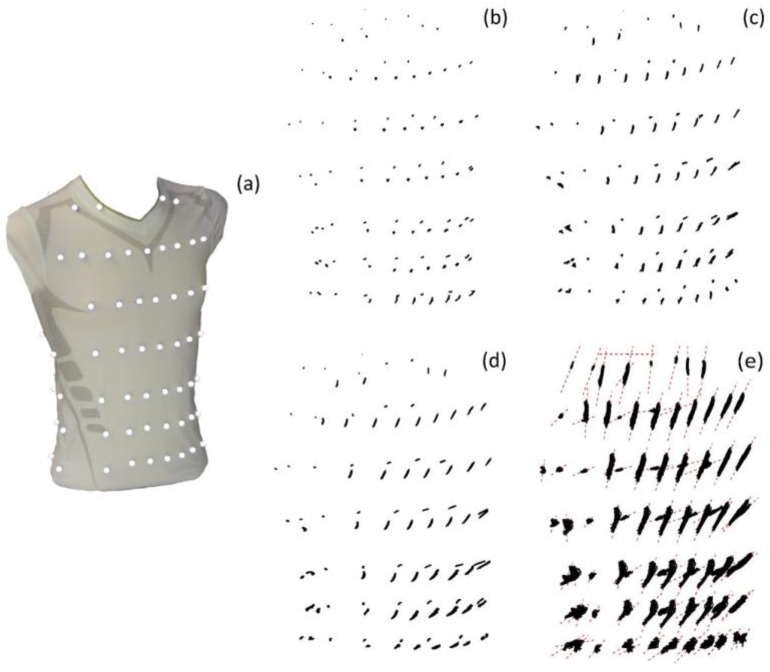
Spatial movement of the MoCap markers on the compression shirt (**a**) during shallow breathing (**b**), normal spontaneous breathing (**c**), medium breaths (**d**) and maximal breaths (**e**), illustrated based on the data of subject 5. The MoCap markers move predominantly on a specific line, which are illustrated in (**e**) as red dashed lines. Figure published in [3].

Motions of the MoCap markers were dimensionally reduced to their major axis using the methods of Laufer et al. [42], as they move predominantly on a specific line (Figure 5). By projecting the marker movements onto their major axis of movement, the dimension of the MoCap data was reduced by a factor of three.

The resulting position changes of all MoCap markers were presented in matrix form (**A_L_**). Two different regression methods (solving **A_L_** x = **v***_spiro_*) allowed for selection of optimal marker subsets of *m* markers. The performances of these subsets was compared with the best marker subset obtained from an exhaustive search of all possible combinations of *m* markers.

The analysis of all possible combinations is a computationally demanding and time-consuming process. However, unlike other methods that can only imply the optimal set based on probabilistic principles, the analysis of all possible combinations provides the best possible and thus optimal set of markers for the determination of the tidal volume. The number (*N*) of all combinations with *k* markers out of a set of *n* markers is given by:

(1) $$N=\left(\begin{array}{c} n\\ k \end{array}\right)=\left(\begin{array}{c} 102\\ 4 \end{array}\right)\approx {5\times 10}^{6},\;N=\left(\begin{array}{c} n\\ k \end{array}\right)=\left(\begin{array}{c} 102\\ 5 \end{array}\right)\approx {8\times 10}^{7}\;\mathrm{or}\;N=\left(\begin{array}{c} n\\ k \end{array}\right)=\left(\begin{array}{c} 102\\ 6 \end{array}\right)\approx {1.4\times 10}^{9}$$

Apart from the analysis of all combinations, regression techniques allowed for faster selection of optimal markers/sensor locations. The first regression technique used was Ridge regression [43], which used a Tikhonov regularization term. Ridge regression finds the argument that minimizes both model error and parameter-squared magnitude (Equation (2)):

(2) $$x_{opt,R}={\left[x_{1},\ldots x_{m}\right]}_{opt,R}^{T}=\underset{x}{\mathrm{argmin}}\left({\left\|A_{L}x-v_{spiro}\right\|}_{2}+\alpha {\left\|x\right\|}_{2}\right)$$

where *m* is the number of chosen parameters and α is the regularization factor of the Tikhonov regularization term *α*ǁxǁ_2_.

For each parameter/MoCap marker of **A_L_,** an argument of **x** was determined. The value of the respective **x** corresponds to the significance of the parameter/the information content of the parameter with respect to the overall system. To reduce the marker set from *n* = 102 to *m* markers, the markers that were assigned the *m* highest absolute values of **x** were selected, because they carried the highest respiratory information of **v***_spiro_*.

The second regression method used was the least absolute shrinkage and selection operator (Lasso) [44], which provided a sparse solution for **x**, and solved:

(3) $$x_{opt,L}={\left[x_{1},\ldots x_{m}\right]}_{opt,L}^{T}=\underset{x}{\mathrm{argmin}}\left({\left\|A_{L}x-v_{spiro}\right\|}_{2}+\lambda {\left\|x\right\|}_{1}\right)$$

where *m* is the number of chosen parameters, and *λ* is the regularization factor of the penalty term of the regularization *λ*ǁ**x**ǁ_1_.

By a suitable selection of *λ*, the number of resulting MoCap markers was reduced to *m*. For comparison, the value of the (Lasso) regularization factor λ was also assigned to the regularization factor α (Ridge).

To increase the significance of this comparison, a bootstrapping resampling procedure was used. Using 16 data sets might be sufficient, but the additional bootstrapping resampling procedure provides more robust evaluation and reduces the relevance of outliers in the data. Hence, for *m* = 4 and *m* = 5, 250 random data segments (of random length) were selected from each of the 16 datasets, on which the analysis was performed separately. To obtain the corresponding data segments, two integer values from a discrete uniform distribution (*randi* function of MATLAB) were used as boundaries of the data segment. For *m* = 6, the number of bootstrapping steps/resampling was reduced to 50 due to time constraints. The analysis was done on a personal computer with a 12th Gen Intel (R) Core (TM) i7-12700K processor with 3.61 GHz (Intel Corporation, Santa Clara, CA, USA) and 64.0 GB RAM (Corsair Gaming Inc., Fremont, CA, USA).

Three MoCap markers along the spine (including the reference marker in the neck) were added to the marker set of *m* markers, chosen by the different approaches. These three MoCap markers/sensors can be used to compensate for non-respiration related movements, such as bending or twisting the upper body.

The final set of *m +* 3 MoCap markers provided by each method allowed for the calculation of the inspiratory volume:

**v***_m_*_+3_ = **A_L_*·*x**_opt_ (4)

In each bootstrapping step, the target number of markers was selected for each of the three different approaches, which yielded a minimal volume error of **v***_m_*_+3_ with respect to **v***_spiro_*. The number of selected markers was according to the size of the targeted subsets of *m* = 4, *m* = 5 or *m* = 6 markers. Each time a MoCap marker was selected (amongst all subjects) the marker was noted. The number of times each marker occurs in all 16 subjects in the selected sensor is analyzed. Finally, the *m* markers with the highest notation amongst all bootstrapping steps and subjects were selected as most valuable MoCap markers.

## 3. Results

**Figure 6:**
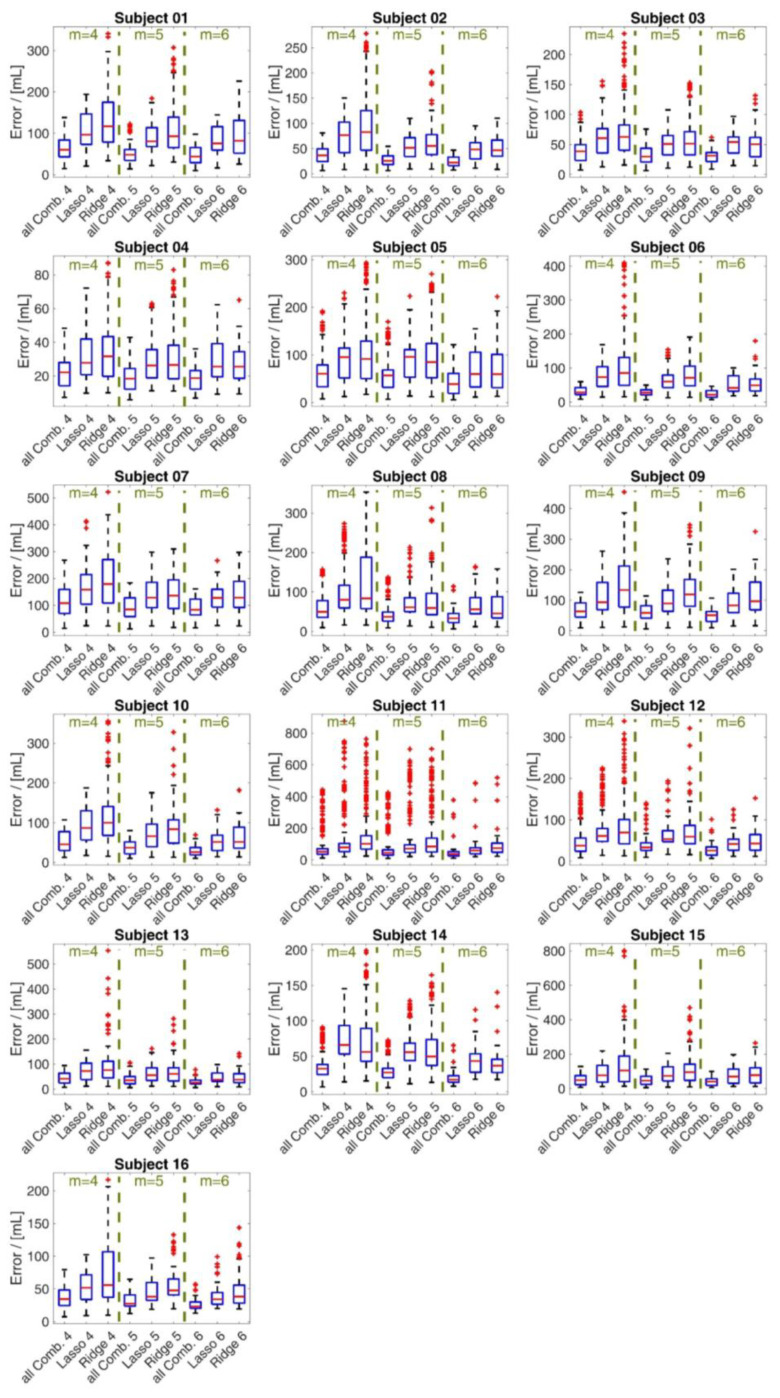
Box plot of the volume errors of **v***_m_*_+3_ for the different approaches related to the spirometer volume **v***_spiro_* during bootstrapping. The errors are shown for subsets of 4, 5 and 6 markers for all 16 subjects. The box and whisker plot illustrate the minimum value, 25th percentile, median, 75th percentile and the maximum value, and the red **+** signs denote outliers.

**Figure 7:**
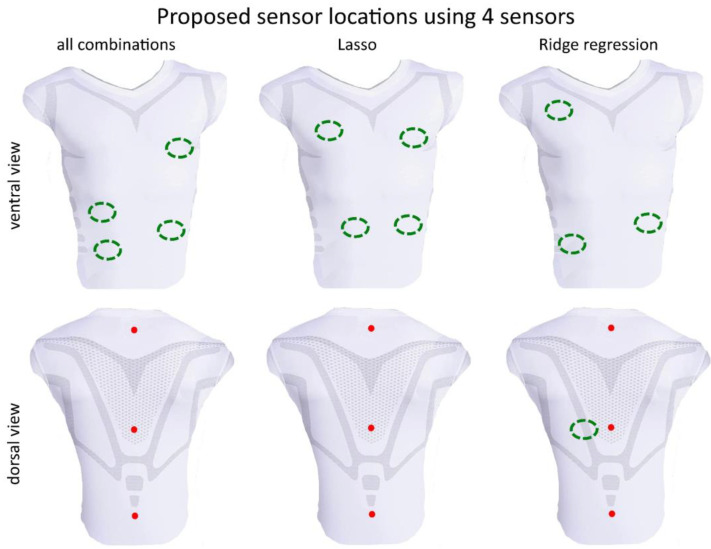
Visualization of the best sensor subset of 4 sensors (green dashed ellipses) by the analysis of all combinations (global optimal subset), Lasso and the Ridge regression–ventral view (**top**) and dorsal view (**bottom**). Red points represent the three datum markers.

**Figure 8:**
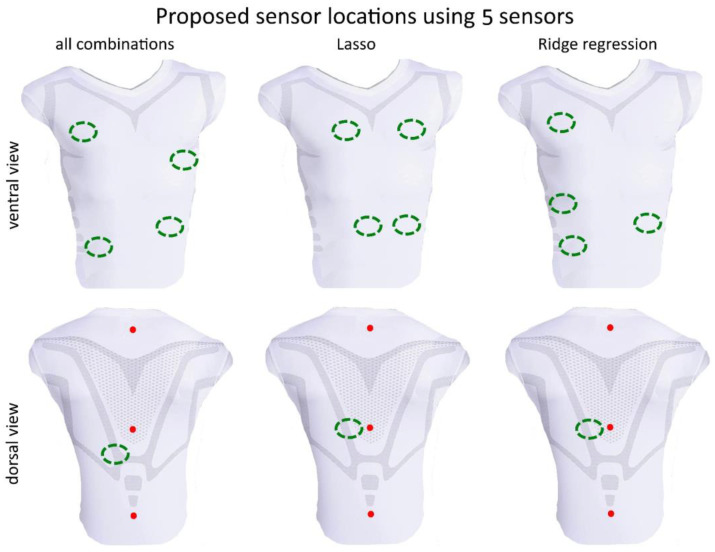
Visualization of the best sensor subset of 5 sensors (green dashed ellipses) by the analysis of all combinations (global optimal subset), Lasso and the Ridge regression–ventral view (**top**) and dorsal view (**bottom**). Red points represent the three datum markers.

**Figure 9:**
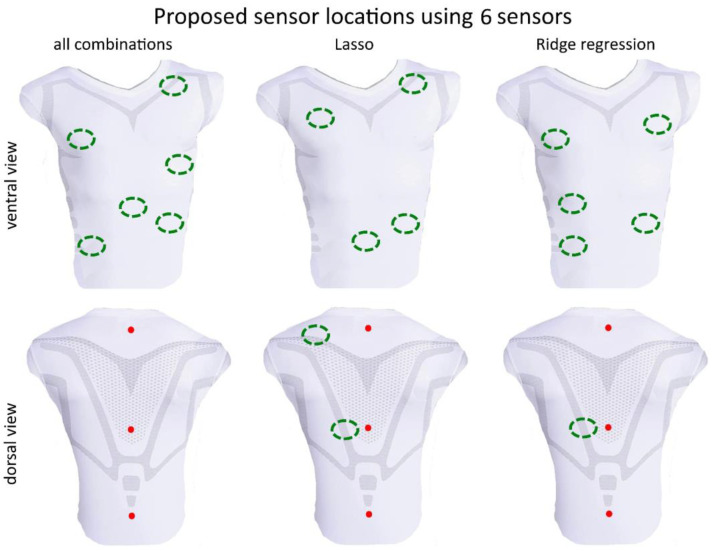
Visualization of the best sensor subset of 6 sensors (green dashed ellipses) by the analysis of all combinations (global optimal subset), Lasso and the Ridge regression–ventral view (**top**) and dorsal view (**bottom**). Red points represent the three datum markers.

For all subjects, each of the 250 (respectively, 50 in a case of subsets of 6 sensors) random segments of measured data during bootstrapping was used to calculate the disparity between the volume estimations from the optimal models from the 3 methods and the gold standard **v***_spiro_* measurement. The resulting errors are illustrated in the box plot shown in Figure 6. The mean values of *λ* respectively *α* are given in Table 3. Based on a random data segment of 60 s, a computation time comparison of the three methods was performed, showing the time savings of the Lasso and Ridge regression regarding the calculation of all possible combinations. This time comparison is listed in Table 4. The sensor positions determined by the different optimization methods for *m* = 4, 5 and 6 are shown in Figure 7, Figure 8 and Figure 9, respectively. In each figure, the added three datum markers along the spine are represented as red points while the *m* optimal locations are indicated in green dashed ellipses.

## 4. Discussion

The use of optoelectronic plethysmography in clinical practice indicates that there is a need for alternatives to respiratory flow measurement via spirometers or body plethysmographs. A smart shirt to measure respiratory volume could be that alternative, would provide convenient measurement and could be used in many clinical scenarios—from sleep apnea monitoring to home care, from respiratory monitoring of comatose patients to exercise monitoring of competitive athletes.

In this study, we investigated the ability of different regression methods to determine the optimal, minimal sensor set that yields accurate inspiratory volume estimation. An optimal method could provide far-reaching support for sensor positioning in smart shirt development [42]. The performed breathing maneuvers (Table 2 and Figure 4) covered a broad range of clinically-relevant tidal volumes. Figure 6 shows the error in estimated tidal volumes of every method. The exhaustive search evaluated all possible combinations and suggested, consequently, the sensor subset that gave the lowest error. Thus, the considerable computational cost was able to yield the global optimal subset (assuming the original marker placement). The errors that occur even for the global optimal subset show that the tidal volumes of the spirometer **v***_spiro_* cannot be reproduced exactly with the chosen number of sensors and limited surface motion information.

**Figure 10:**
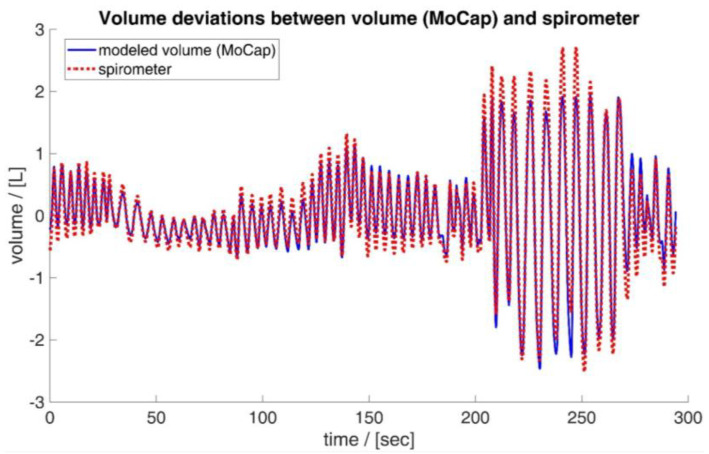
Deviations between the volume obtained by the MoCap system (blue) and the spirometer (red). Maximal deviations occur at maximal breaths—exemplarily illustrated based on the data of subject 15.

In a previous study [45], deviations of tidal volumes measured by an optoelectronic plethysmography device from **v***_spiro_* were observed mainly for larger breaths. These deviations might be caused by pressure-induced compressions of the air in the thorax, whereas these compressions do not influence the flow measurement via the spirometer. These deviations are exemplarily shown based on the data of subject 15 in Figure 10. Apart from the maximum deviations, this seems to be mainly an underestimation of volumes, which could be caused by filtering effects (transfer function).

**Figure 11:**
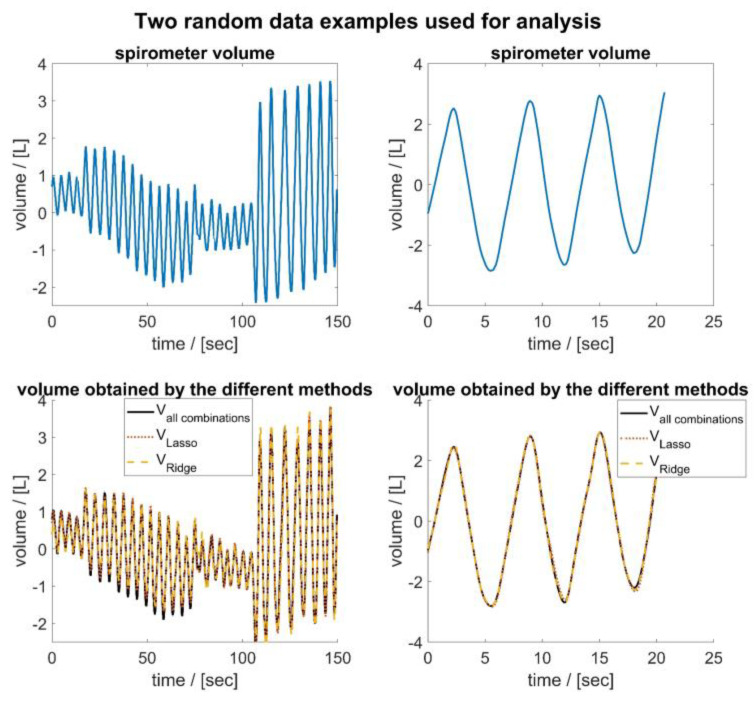
Two randomly selected areas of the data used for analysis during bootstrapping, illustrated based on the data of subject 5 and for *m* = 4. The upper part shows the volume curve of the spirometer, while the lower part shows the corresponding volume obtained by the three different methods.

Expectedly, involving higher number of sensors enhances measurement of tidal volume and reduces the error (Figure 6), since any added information would improve a regressive estimation. Due to the bootstrapping process used to artificially expand the data set, the actual performance may differ slightly from the actual performance. In this respect, shorter data segments can lead to smaller errors because they typically have fewer divergent trends and features in respiratory curves and can be better fitted to **v***_spiro_*. Figure 11 shows exemplarily two data segments of random length that were used for analysis during bootstrapping. It can be seen that shorter data segments have fewer divergent trends and features. In addition, it can be seen that the volumes determined via the movements of four MoCap markers/sensors are highly correlated with **v***_spiro_*, independent of the method used for selection of the MoCap markers/sensors.

Overall, the mean errors (Figure 6) of the two regression methods are of similar magnitude. The Lasso’s mean errors in the case of *m* = 4 were slightly lower for 14 of the 16 subjects and in case of *m* = 5 for 13/16 subjects compared to the mean errors of the Ridge regression. For *m* = 6, the Lasso’s mean errors were lower in 7/16 subjects and nearly equal in 5/16 subjects.

While in the Lasso, the value of *λ* was determined by the number of desired markers/sensors, the mean error of the Ridge regression can be influenced by the choice of *α*. A small α leads to smaller errors of the regression term ${\left\|A_{L}x-v_{spiro}\right\|}_{2}$ while a larger *α* leads to faster convergence of the calculation. Thus, the choice of α affects the mean error of the calculation. Since *α* was assigned the value of *λ* in this study, a comparison is possible, but it is only indicative and should not be the decisive criterion for the choice between the Ridge and Lasso, as a change in the regularization factors may influence the determined errors.

Interestingly, the Lasso’s mean errors as well as the Ridge’s mean errors were consistently higher than the mean errors of the global optimal set in all cases. Compared to the Lasso and the global optimal subset, the Ridge regression has higher peak errors and higher deviations for nearly all subjects. Figure 6 indicates that the Ridge regression is much more susceptible to yielding outlier high errors than the Lasso regression. The results of this study are in agreement with the outcomes of Ng et al. [46] since in our case the Lasso method (L_1_ norm) also showed clear advantages in terms of errors, robustness and outliers compared to the Ridge regression (L_2_ norm). The Lasso approach is known to produce a sparse solution, prevent overfitting and remain robust to outliers. All these features of the Lasso method are advantageous when selecting sensors from a sensor set. In particular, the sparse solution supports the selection of the smallest possible subset, which reduces complexity, error-proneness and cost. In particular, the Lasso shows tremendous advantages in the investigation of respiration-induced upper body movements. Upper body movements during respiration show a high correlation with the respiratory volume itself. The deeper a subject inhales the more the upper body expands. The high correlations of the movement parameters with the respiratory volume also imply high correlations of the respiratory parameters with each other. When the Lasso method is applied to data that are highly correlated with each other especially, it works well and provides a sparse solution.

The Ridge regression does not necessarily provide sparse solutions; on the contrary the regression is reducing the difference in the measurement positions and thus complicates the selection. However, the savings in both time (Table 4) and computational costs compared to the exhaustive search is considerable. The computational demand is reduced from an exponential effort for a full exhaustive search down to the order of *O* (*n*^2^) for the regression methods. Thus, in general, the comparatively light computational burden of the Ridge regression implies it has its legitimate benefit in some situations.

For a smart shirt as a medical product, an exhaustive search, although computationally intensive, provided significantly lower volume errors while the regressive models provided more consistent sensor sets. In practice, the maker location from such an analysis would be fixed and constant across patients. The regressive models could then be used to produce precise estimations of inspiratory volume. However, if more precise respiratory measurements were possible, the regression could be adapted to develop an individualized model for precise and accurate estimations of the inspiratory volume.

While there is general agreement in the optimal positions of MoCap markers/sensors in a smart shirt across regression methods, the regression methods did not yield exactly the same positions. It appears that the Ridge regression agrees with the positions of the best combination slightly more consistently than the Lasso. The positions obtained from the Ridge regression and exhaustive search are more lateral in the ventral region than the positions obtained by the Lasso. This is also evident for *m* = 5 (Figure 8) and *m* = 6 (Figure 9), where the Lasso method selects an area for a sensor in the central abdominal region.

When increasing the number of sensors from *m* = 4 (Figure 7) to *m* = 5 (Figure 8) then *m =* 6 (Figure 9), the previous positioning remains to a large extent consistent, usually only one marker/sensor is added, and the previous selection (*m*−1) is preserved. In particular, the Ridge regression consistently reproduces previously selected positions. The Lasso method shows small shifts of the previously selected areas. The analysis of all combinations shows in this respect the biggest differences. The areas selected for smaller *m* are only partially preserved for larger *m*. Some sensors are no longer selected at all. For example, the dorsal region at *m* = 5 (Figure 8) is no longer selected when *m* is increased. This marker/sensor might be irrelevant due to the three fixed included sensors/datum markers along the spine.

Since the sensor positions did not differ significantly amongst the evaluated methods, both regression methods can support the development of smart shirts for respiratory volume estimation. For larger sensor subsets, the complex and exhaustive search is no longer possible and methods, such as the Ridge/Lasso, must be used.

**Figure 12:**
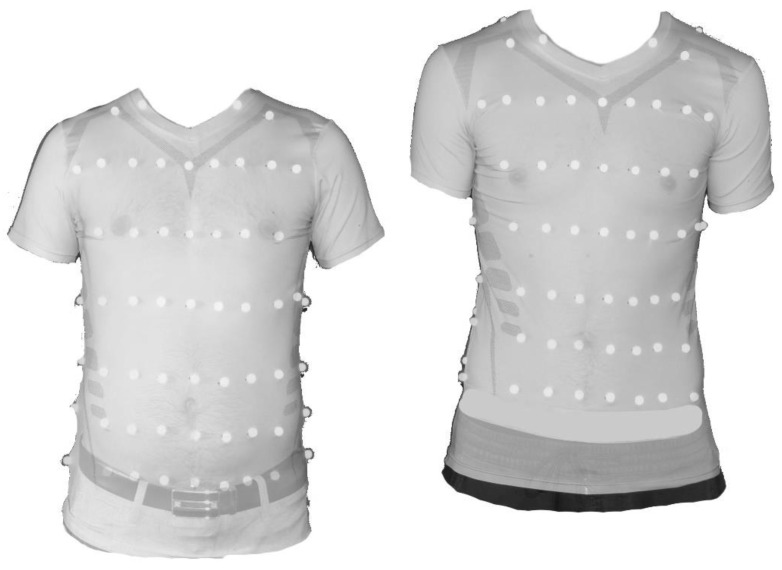
Illustration of variations in anatomical marker positions for subjects of different body shapes (visualized via subject 3 (**left**) and subject 1 (**right**)).

There were some limitations in this study. One limitation was that the size of the shirt led to some error. This was clearly observed between tall and short participants (Figure 12). In particular, the positions of the markers were in slightly different positions on the upper body due to the uniform compression shirt and were not exactly in the same place. However, this error would also occur in a smart shirt, as the shirt is not individually tailored to the particular subject. Different shirt sizes can limit this error within certain limits; however, a tight fit of the shirt is essential in this context. Despite this fact, the results remained within acceptable bounds for these subjects.

Another limitation was that the shirt could slip on the skin surface during respiration [47]. This error was only observed with very large breaths. Therefore, it represents a systematic error, which cannot be avoided without disturbing the subject. It would be possible to reduce the shirt-to-skin movement with a very tight fit of the smart shirt and/or an adhesive or clinging inner material in the shirt. However, such an approach may not improve results sufficiently to justify the irritation the subject may feel as a result of the adhesive. Multiple tissue interfaces exist between the alveoli and the skin surface, and these shift against each other during respiration. Adding another layer to the skin–shirt interface seems unlikely to be a confounding factor.

Further measurements with more subjects of different ages and body shapes should confirm the results of this study and provide better insights into the systematic nature of sensor selection. With more subjects, a subgroup analysis would also be possible (for example, to examine the effects of different breathing patterns, such as abdominal or chest breathing). Most participants in our study were male (13/16) and young adults (13/16 ≤ 30 years) in the healthy BMI range (15/16). This leads to bias that must be corrected prior to clinical application. A study with subjects with a lung disease, such as chronic obstructive pulmonary disease or cystic fibrosis, could provide further insight into optimal sensor selection. Additionally, extremely lung-sick patients who have only a small portion of their lung capacity available might be outside the range of tidal volumes covered by our subjects. In the case of subjects with lung disease, other aspects could play a decisive role that is not apparent in the case of lung-healthy subjects. In particular, the current study used subjects who did not have significant asymmetry in their pulmonary filling. In contrast, individuals with cystic fibrosis or other lung diseases may have significant asymmetries with respect to the left and right sides of the thorax and abdomen.

## 5. Conclusions

This study shows that the selection of sensors with linear regression depends on the regression method itself. The Lasso method is preferable to the Ridge regression because it provides both more robust and sparser solutions. However, both regression methods have their justification in this field of application, as they significantly reduce computation time and effort but with the disadvantage that their performance suffers compared to the performance of the optimal subset.

Both regression methods can support smart shirt development for respiratory volume estimation by guiding the type and optimal location of the required sensors. A smart shirt for respiratory volume estimation could replace spirometry and would allow for a more comfortable and long-term measurement of respiratory parameters in homecare or clinic.

## Figures and Tables

**Table 1:** Details of the Participants. Table published in [3].

Subject	Height/[m]	Weight/[kg]	BMI/[kg/m^2^]	Age/[years]	Gender
1	1.84	75	22.15	18	male
2	1.72	65	21.97	19	female
3	1.70	56	19.38	26	male
4	1.67	57	20.44	18	female
5	1.83	78	23.29	30	male
6	1.75	70	22.86	32	male
7	1.79	75	23.41	53	male
8	1.74	63	20.81	20	male
9	1.70	68	23.53	24	male
10	1.82	73	22.04	30	male
11	1.74	81	26.75	31	male
12	1.73	67	22.39	19	male
13	1.71	60	20.52	23	male
14	1.68	66	23.38	21	female
15	1.88	75	21.22	20	male
16	1.83	82	24.49	28	male

**Table 2:** Performed respiratory patterns. Table published in [3].

Pattern Number	Duration [s]	Breathing Pattern
1	30	spontaneous breathing (normal)
2	60	shallow breathing
3	30	spontaneous breathing (normal)
4	60	medium breaths
5	30	spontaneous breathing (normal)
6	60	maximal breaths
7	30	spontaneous breathing (normal)

**Table 3:** Mean values of the regularization factors *λ* (Lasso). The regularization factor *α* (Ridge) was set to the value of *λ*.

m	Mean (λ)
4	0.096
5	0.059
6	0.036

**Table 4:** Average calculation time required by Ridge regression and Lasso compared to the average time needed to analyse all combinations based on random data segments of 60 s.

	Average Required Time [s]		
*m*	Ridge Regression	Lasso	All Combinations
4	0.037	0.066	81
5	0.046	0.073	1573
6	0.047	0.112	46,886

## Data Availability

The data presented in this study are available on request from the corresponding author.

## Abbreviations

The following abbreviations are used in this manuscript:

Cx	Cervical vertebrae x (1 ≤ x ≤ 7)
Lasso	Least absolute shrinkage and selection operator
Lx	Lumbar vertebrae x (1 ≤ x ≤ 5)
MoCap	Motion capture system
Tx	Thoracic vertebrae x (1 ≤ x ≤ 12)
**v**_*m*+__3_	Volume obtained via the selected MoCap marker subset of *m* MoCap markers and the additional 3 MoCap marker along the spine
**v***_spiro_*	Volume obtained with spirometer
